# “It is like an umbrella covering you, yet it does not protect you from the rain”: a mixed methods study of insurance affordability, coverage, and financial protection in rural western Kenya

**DOI:** 10.1186/s12939-023-01837-2

**Published:** 2023-02-06

**Authors:** Beryl Maritim, Adam D. Koon, Allan Kimaina, Cornelius Lagat, Elvira Riungu, Jeremiah Laktabai, Laura J. Ruhl, Michael Kibiwot, Michael L. Scanlon, Jane Goudge

**Affiliations:** 1 Consortium for Advanced Research Training in Africa (CARTA), Nairobi, Kenya; 2 Centre for Health Policy, School of Public Health, University of the Witwatersrand, Johannesburg, South Africa; 3 Academic Model Providing Access to Healthcare (AMPATH), Eldoret, Kenya; 4 Department of International Health, Johns Hopkins Bloomberg School of Public Health, Baltimore, USA; 5 School of Medicine, Moi University, Eldoret, Kenya; 6 School of Medicine, Indiana University, Indianapolis, USA

**Keywords:** Affordability, Health insurance, Informal workers, Rural, Kenya

## Abstract

Countries in Sub-Saharan Africa are increasingly adopting mandatory social health insurance programs. In Kenya, mandatory social health insurance is being implemented through the national health insurer, the National Hospital Insurance Fund (NHIF), but the level of coverage, affordability and financial risk protection provided by health insurance, especially for rural informal households, is unclear. This study provides as assessment of affordability of NHIF premiums, the need for financial risk protection, and the extent of financial protection provided by NHIF among rural informal workers in western Kenya.

**Methods** We conducted a mixed methods study with a cross-sectional household survey (*n* = 1773), in-depth household interviews (*n* = 36), and 6 focus group discussions (FGDs) with community stakeholders in rural western Kenya. Health insurance status was self-reported and households were categorized into insured and uninsured. Using survey data, we calculated the affordability of health insurance (unaffordability was defined as the monthly premium being > 5% of total household expenditures), out of pocket expenditures (OOP) on healthcare and its impact on impoverishment, and incidence of catastrophic health expenditures (CHE). Logistic regression was used to assess household characteristics associated with CHE.

**Results** Only 12% of households reported having health insurance and was unaffordable for the majority of households, both insured (60%) and uninsured (80%). Rural households spent an average of 12% of their household budget on OOP, with both insured and uninsured households reporting high OOP spending and similar levels of impoverishment due to OOP. Overall, 12% of households experienced CHE, with uninsured households more likely to experience CHE. Participants expressed concerns about value of health insurance given its cost, availability and quality of services, and financial protection relative to other social and economic household needs. Households resulted to borrowing, fundraising, taking short term loans and selling family assets to meet healthcare costs.

**Conclusion** Health insurance coverage was low among rural informal sector households in western Kenya, with health insurance premiums being unaffordable to most households. Even among insured households, we found high levels of OOP and CHE. Our results suggest that significant reforms of NHIF and health system are required to provide adequate health services and financial risk protection for rural informal households in Kenya.

## Introduction

There has been an increasing focus on achieving universal health coverage (UHC) with international calls to extend coverage of financial protection and the availability of quality health services to all people. For most low- and middle-income countries (LMIC), out-of-pocket (OOP) spending remains the dominant form of financing healthcare [1, 2]. It is estimated that OOP spending on health causes 150 million people to suffer financial catastrophe and pushes 100 million people globally into poverty every year [3]. In a recent analysis of 36 sub-Saharan African (SSA) countries, only four had health insurance coverage rates above 20% (Rwanda, Ghana, Gabon and Burundi) and insurance coverage was highly skewed towards higher income households and those in the formal economy [4].

Countries in SSA are increasingly turning to mandatory social health insurance systems to increase coverage [1], and several have implemented or are in the process of implementing some form of mandatory health insurance programs [5, 6]. A mandatory health insurance program is one in which members and contributions are demanded by law from a certain set of people or the entire population and is often accompanied by efforts to subsidize poorer households [1, 7]. Although declared compulsory, most “mandatory” insurance schemes cover households in the formal sector only while informal worker membership is voluntary in part due to weak mechanisms to enforce insurance mandates [8]. In Kenya, the national social health insurer, National Hospital Insurance Fund (NHIF) has been mandatory for both formal and informal sector workers since the NHIF Act of 1998 [9], and reinforced by amendments to the Act in 2022 that underscore mandatory membership [10].

Kenya has undertaken numerous health insurance reforms through NHIF aimed at increasing coverage among informal workers while also ensuring financial viability of the insurance program. Recent reforms include the expansion of the benefit package to include both inpatient and outpatient services; flexible monthly contributions; introduction of easy payment methods such as mobile money payments; and an upward revision of premiums to finance the expanded benefit package [11]. Monthly NHIF insurance premiums for informal workers increased significantly from Kenya Shilling (Ksh) 160 (US$1.3) to Ksh 500 (US$4) in the year 2015 raising questions about the affordability of health insurance for poorer households [11, 12]. The government has established various subsidy programs to promote access to reproductive, maternal and child health services among pregnant women (Linda Mama program); provide coverage to indigent households (national indigent program), older persons and persons living with severe disabilities (health insurance subsidy program); and free cover for secondary school students (Edu-Afya program) [13]. Despite these efforts, inequitable and low coverage of insurance among informal workers in Kenya persists with only 8% enrolled in health insurance [14].

Increasing enrolment and coverage in health insurance programs for informal sector workers and their households, who constitute a majority of the population in many LMICs, is critical to advancing UHC. Currently, insurance schemes face several key challenges to expanding coverage of informal workers, with programs experimenting with subsidies or waivers for households that cannot pay, progressive premium pricing, and strengthening mechanisms for health insurance enrolment and payment [5]. To guide these efforts, additional data is needed to better understand health insurance enrolment and coverage among the informal workers, including affordability of insurance premiums and the protection from financial risk provided by insurance coverage.

Kenya represents an important case study as it has consistently attempted to expand financial protection through NHIF in its quest for UHC. This includes a failed 2004 attempt at dramatically expanding NHIF’s remit by moving toward comprehensive social health insurance [15]. Smaller, incremental reforms in the 2010s improved the coverage, scope, and financial stability of NHIF [11]. Furthermore, NHIF plays a central role in recent national policy strategies like Vision 2030 and the “BIG four” agenda that aims to achieve UHC by 2022 [16, 17]. Yet, to accomplish this, NHIF will have to expand health insurance coverage to the informal sector, something it has struggled to do in the past. For this reason, more research is needed to understand the barriers NHIF faces in reaching this large and vulnerable segment of the population. In this paper, we describe a mixed methods study that assessed health insurance coverage among the rural poor in western Kenya, including the affordability of health insurance premiums and the burden of health expenditures among insured and uninsured households.

## Methods

### Study design

We conducted a cross-sectional mixed methods study using household survey, in-depth interviews (IDI) with household respondents, and focus group discussions (FGD) with community stakeholders [18]. The study was embedded in a larger household survey study conducted between August and October 2021 by investigators at the Academic Model Providing Access to Healthcare (AMPATH), an academic collaboration between Moi University, Moi Teaching and Referral Hospital, and a consortium of universities led by Indiana University [19].

### Study setting

Data was collected from households in Bunyala sub-county, with a total of 18,229 households, in Busia county in Kenya, located on the western border with Uganda where AMPATH implements a population health program [19]. The UHC pilot site was chosen because of longstanding partnership between AMPATH and Busia County leadership implementing health programs in the region and disproportionate number of rural informal households and high levels of poverty. This study was embedded on baseline survey to inform roll out of UHC pilot implemented by the County Government of Busia in partnership with NHIF and AMPATH.

### Study participants

We utilized a program database that included all households in Bunyala sub-county to randomly select households to participate in the survey. The strata for our sample were made up of six administrative units called locations with an average of 3,038 households per location., Locations are the second smallest administrative unit after sub-locations. A household list was created from the database and used to randomly select households that would be invited to take part in the household survey under each stratum. Eligible household respondents were those aged 18 years and above and who reported having sufficient information about household spending and healthcare use for themselves and other household members. Interviewers made 3 attempts to reach a household representative to participate. Households that were not available for interviewing either resulting from being absent or refusal on the third attempt were excluded from the survey and considered a non-response. A total of 1,773 households took part in the quantitative survey.

For qualitative interviews and FGDs, we identified a sub-sample of households that participated in the household survey. From this group, for each of the 6 locations, we purposively chose two households from each of the following two groups to participate in IDIs: those who are currently enrolled in the NHIF, those who have previously enrolled in the NHIF but are no longer enrolled, and those who have never enrolled in the NHIF. We also purposively recruited 6–8 community stakeholders for FGDs. These included opinion leaders, local administration, civil society organization members, community health volunteers (CHVs), ward administrators, youth representatives, teachers and religious leaders identified based on their active advocacy on health insurance related issues. We conducted 6 FGDs, one in each of the 6 locations with a total of 49 participants (30 males and 19 females) (Table 1)

**Table 1:** Data collection method and study participants

Interview method	Number of interviews	Participants
Household survey	1,773	Household respondents (˃18 years)
In depth interviews	36	Household respondents (˃18 years)
Focus group discussions	6 groups with a total of 49 participants (30 male and 19 female)	Community stakeholders/representatives

### Data collection

#### Household survey

A structured survey questionnaire was used by trained interviewers to collect individual and household level data, including household size, education level, health insurance status, health status, marital status, income level, healthcare spending and utilization, and health status. Local government representatives assisted in identifying data collectors who had prior knowledge in collecting data for national surveys. Interviewers received training by the study team on survey concepts, practices, and guidelines and using a computer tablet for data collection. The survey team worked with CHVs to approach household representatives at regularly scheduled visits to minimize disruptions and increase participation. The interviews were confidential and the CHVs did not participate in the interview process.

#### In-depth interviews and focus group discussions

IDIs were performed in-person at respondents’ houses while FGDs were conducted at the nearest health facilities or community space by members of the study team trained in qualitative interviewing. Interviews and FGDs were conducted using a semi-structured interview guide designed to elicit household illness histories, experience with health insurance (or lack thereof), healthcare spending and affordability and coping strategies for accessing healthcare services. Each interview lasted 30–40 min on average while FDGs were between 2–2.5 h. Debriefing sessions in between interviews and FGDs were held periodically between BM, JG and ADK to improve the interviewing process and identify emerging themes to probe.

### Data analysis

#### Quantitative analysis

Descriptive statistics were calculated for household participants, including household size, education, marital status, income level, health status, and healthcare spending and utilization. Health insurance status was self-reported and households were categorized into insured and uninsured. Using these survey data, we calculated the affordability of NHIF premiums, OOP spending on healthcare and its impact on impoverishment, and incidence of catastrophic health expenditures (CHE) and household factors associated with CHE. Definitions and methods to calculate these outcomes are provided below.

##### Affordability of health insurance

To estimate the affordability of NHIF’s premium rate of Ksh 500 per month for a household among uninsured households, we expressed the amount of the insurance premium as a percentage of the total monthly household expenditure among the uninsured. We set affordability the threshold at 5% as this is the standard benchmark for affordability in the literature [20].

##### OOP and impoverishment

OOP were defined as household healthcare costs including payments for direct medical services such as medications, medical fees, hospital / clinic fees, laboratory diagnostic tests and indirect healthcare-related costs including transportation costs to and from health facilities. We excluded costs covered by health insurance from OOP healthcare costs. We calculated total OOP on healthcare as a percentage of households’ annual expenditure. We disaggregated OOP by household insurance status and whether OOP spending was for inpatient versus outpatient services.

We adopted the Kenya national poverty line of consumption expenditure of Ksh 3,252 (US$ 29.6) for rural areas per individual per month in order to assess the impact of OOP on poverty levels [21]. Poverty levels (per capita) were calculated before and after OOP. We present three widely accepted measurements [22]: (1) the poverty household head count, which measures the percentage of households below the established poverty line; (2) the poverty gap, which measures the total deficit from the poverty line; and (3) the normalized poverty gap, which is calculated by dividing the estimated poverty gap by the established poverty line. The normalized poverty gap is helpful for cross-national comparisons between nations with various poverty thresholds and monetary systems.

##### Catastrophic health expenditures

To calculate CHE, we expressed total household OOP as a share of total non-food household expenditure over one year. We defined CHE as when OOP were at or above 40% of non-food expenditure as established elsewhere in the literature [11]. The incidence of CHE is represented by the proportion of households that had to pay for catastrophic medical expenses [22]. Despite estimating the percentage of families who experience CHE, the catastrophic head count does not provide information on the severity of the catastrophe (i.e., by how much a households’ OOP payment exceeds the catastrophic threshold). To estimate severity of CHE, we calculated a catastrophic overshoot defined as the average amount by which OOP payments, as a percentage of overall expenditure, exceed the catastrophic threshold. An average catastrophic overshoot was computed for all the sampled households.

We used bivariate and multivariate logistic regression to identify household factors associated with incurring CHE where the dependent variable was binary – i.e., a household incurred CHE or not. We used odds ratio (OR) and adjusted odds ratio (AOR) to report associations between household factors on the probability of incurring CHE. We incorporated variables that were significant at *P*-value < 0.05 into a multivariable logistic regression model and included variables that we purposively selected based on the literature even if they were not significant in the bivariate analysis [23]. Confidence internals of 95% are reported and significance level α = 0.05 was used.

#### Qualitative analysis

Audio recordings from the IDIs and FGDs were translated and transcribed verbatim by trained transcribers. Data were analysed using a thematic framework analysis approach [24]. One author (BM) listened to audio recordings and read and reread transcripts to check for accuracy of the transcripts and identify preliminary themes and codes. Two additional authors (ADK and JG) read a sample of transcripts and agreed on a coding framework in an iterative process. One author (BM) coded transcripts using both inductive and deductive codes utilizing the NVIVO 12 analysis software. Repeating concepts emerging from the interviews were grouped into basic themes. Basic themes were then grouped into organizing themes in an iterative process, aimed at drawing the relationships between categories of organizing themes. Findings were interpreted based on connections between various themes and accompanied by supportive quotes from the interviews. Illustrative household cases were included to further contextualize participants’ experiences and the interconnectedness of themes.

## Results

### Household and respondent characteristics

A total of 1,773 households, representing 8,610 individuals, took part in the household survey. The average household size was 5 members. Sixty-four percent of the respondents were female and the average age of the respondents was 47 years.

Many households reported facing significant financial hardship. They struggled to meet basic household needs like food, housing and education. They reported going without meals, children being delayed joining school, being sent back from school while others did not proceed to tertiary institutions. Financial hardship was compounded during the study period by severe floods and other climate-related phenomena, socioeconomic consequences of the COVID-19 pandemic. Some of the respondents lived in regions periodically affected by floods caused by heavy rainfall and the overflow of Lake Victoria, affecting farming and fishing activities in the area.

“*We were dependent on Lake Victoria but right now fish quantities are small. I also used to engage in farming where I planted pawpaw, sweet potatoes and bananas but after the floods took away everything, I am back to zero*.” IDI_B_Active_02.

Some respondents attributed their hard economic conditions to the COVID -19 pandemic that caused a substantial economic burden on households. One respondent reported worsened economic conditions during the pandemic resulting in significant drop in household income.

“*We have really struggled, especially last year [2020]. There are no jobs, corona is here, and there is a lockdown that even made it worse*” IDI_A_Never_01.

### Health insurance coverage and affordability of health insurance premiums

The NHIF insurance premium would amount to more than 5% of household expenditure for 60% of uninsured households and 80% of insured households. Only 11.6% of the households reported having any form of health insurance with 91% of insured households reporting NHIF as their insurer. Among those without health insurance, 79% of the respondents cited the high cost of premiums as the main reason for lacking NHIF.

Participants expressed that NHIF premiums (Ksh 500 or US$4 monthly) were too high. Many households indicated they could not afford to enrol or keep up with the premiums as they would have to decide between meeting more pressing household needs or paying for insurance.

“*Perhaps I have earned Ksh 1,000 or Ksh 500 for this month; will I go to pay NHIF and my kids to sleep hungry-No? They should put an amount where the low-level people can pay without struggling.*” Respondent 3_FGD D.

Additionally, those who had defaulted from NHIF (i.e., failed to make monthly payments) also expressed concern that the additional penalties charged for defaulting were too high and were a barrier to re-activating their insurance. Vulnerable households such as widowed households and single parent homes particularly found it hard to keep up with paying monthly premiums and reported defaulting on payments to NHIF.

Both insured and previously insured respondents noted that the full cost of healthcare was often not covered by NHIF, with NHIF perceived to offer better coverage of inpatient costs compared to outpatient services. Unsurprisingly, many respondents with NHIF reported enrolling because of the presence of a chronic illness in the household requiring treatment. Anticipated medical procedures was also found to be a driver of health insurance enrolment as demonstrated by the case of one respondent, Cleophas (Case study 3). He enrolled for insurance because a healthcare provider advised him so that his frequent medical costs and the cost of an anticipated operation would be covered by NHIF. Despite numerous challenges he and his household have experienced using the cover, he continues to pay for the cover. Cleophas compares having NHIF to ‘*having an umbrella covering you yet it does not protect you from the rain’*.

**Table:** 

**Case study Household 3: Under coverage**
Cleophas is a farmer and his wife (56 years old) have four children-one in college and three children of high school going age. They also take care of three orphans aged 12, 10, and 7 years. He has NHIF and his wife and biological children are all included in his cover. Cleophas has had two hernia operations costing Kshs. 100,000(US$830) and Kshs. 81,000 (US$675) each. Both times, he fundraised for the hospital bills through friends, family and well-wishers. He also sold some cows to raise additional funds. It is then that he decided to enroll for NHIF because a provider advised him to get insurance to avoid incurring more high medical bills for future treatment. Recently, one of his daughters fell sick while in boarding school away from home and he had to send Ksh. 5,000(US$41) for her treatment even though she is covered under his NHIF plan. The outpatient facility she is registered under is closer to home but farther from her school and so they had no choice but to pay cash. Although she should have been covered under the Edu-Afya program that provides health insurance to all secondary students, she had not been registered through the school at the time. In the past, the family has used chama (community financial groups) loans to pay for healthcare. Despite having NHIF, Cleophas reports paying for many test and scans. He complains about the high cost of premiums but continues to pay because he needs to access treatment and procedures that are otherwise too costly but wishes the challenges with the cover would be addressed so he can be fully covered. (IDI_A_Active_02)

There were also concerns that the poor services offered at health facilities discouraged many people from enrolling for insurance because they would still have to pay OOP for medication at private chemists.

“*First of all, that amount is very high. 500 shillings is very high. Secondly, there are no drugs in the hospital, so insurance doesn’t help me. It is better for me to just go to the chemist and buy drugs. I don’t see the importance of NHIF*.” IDI_A_Not_Active_01.

Public primary health facilities that were supposed to offer free health services often lacked medication and supplies. A few respondents reported only visiting the facility for diagnosis and proceeding to private chemists to buy prescribed medication. Accredited health facilities were located far and increased the cost of seeking care because of transport to the facility. Unsurprisingly, the quality of health services was also cited as a reason for not seeking care among the uninsured.

Public facilities were also noted to have frequent healthcare worker strikes that interrupted service provision in these facilities. These conditions forced the household to not seek care or to go to private or mission facilities making the cost of seeking care even higher. The pandemic also disrupted healthcare utilization because some of the healthcare facilities were converted to isolation centres.

“*When this skin condition recurred, I could not go back to the hospital because it was an isolation facility for those affected by corona and they did not provide care to other people as much*” IDI_A_Active_01.

#### Healthcare utilization

Illnesses in the four weeks preceding the survey were reported for 3,070 (32.1%) individuals, of which 1,137 (37%) did not seek care. Self-medication was cited by a majority of the respondents as the reason for not seeking care (48%) while the second leading reason was lack of money to pay for healthcare (24.6%) (Table 2). The reasons for not seeking care were similar across insured and uninsured households.

**Table 2:** Reasons for not seeking care by insurance status

Reason for not seeking Care	*Uninsured* % (n^a^)	Insured % (n^a^)
Self-medication	48(616)	46.7(529)
Lacked money	24.6 (316)	26.7 (302)
Illness not considered serious enough	16.9 (217)	16.1 (182)
Long distance to provider	2.7 (34)	2.6 (30)
Drugs not available at the health facility	2 (26)	1.9 (22)
Poor quality service	1.1 (14)	1.0 (11)
Fear of discovering	0.9 (11)	1.0 (11)
High Cost of Care	0.8 (10)	0.7 (8)
Terminal illness	0.5 (6)	0.5 (6)
Religion /cultural reasons	0.2 (3)	0.3 (3)
Other	2.3 (30)	2.6 (29)
**Total**	**100 (1283)**	**100 (1133)**

^**a**^Responses based on individual illness episodes and respondents could cite multiple reasons for not seeking care

Healthcare seeking decisions were often based on perceived affordability and source of funds. Many reported not seeking care when they were sick, going to the nearest health facilities to avoid high transport costs, postponing medical procedures, avoiding private or ‘big’ facilities which were perceived to be expensive and using herbal medicine:

“*The doctor prescribes you medicine and tells you to go buy but you don’t have that money. You will just hold on to the painkillers that maybe you are given free at the hospital so you can buy some sugar and rice and the children can get something to eat. You just survive by God’s grace*.” IDI_A_Never_01.

Most respondents opted to first buy drugs at local private chemists or shops and only proceeded to seek attention at the health facility if the condition got worse.

#### Incidence of OOP

**Fig. 1:**
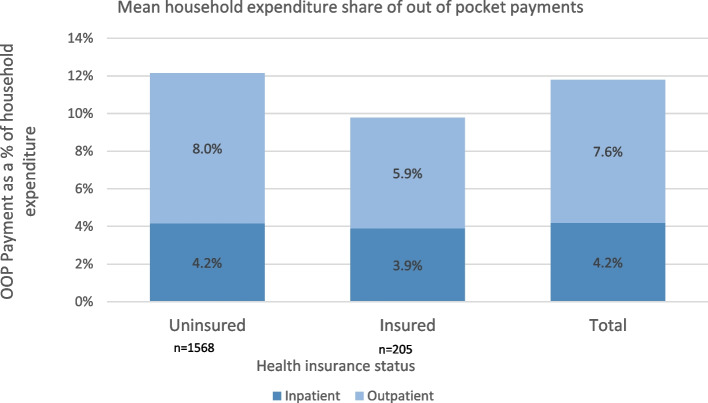
Mean household expenditure share of out-of-pocket payments by health insurance status

Figure 1 shows mean household budget share of OOP payments by health insurance status. Overall, households spent 8% of their annual expenditure on outpatient services and 4% on inpatient services. Mean total OOP payments amounted to 12% of households’ annual expenditures. Uninsured households spent a slightly larger share of their expenditures on healthcare (12%) compared to insured households (10%). However, average monthly OOP spending for the insured households (Ksh. 9,602(US$80)) was slightly higher than the uninsured (Ksh. 7,053(US$59)). Outpatient care constituted a higher share of expenditures compared to inpatient care, with the uninsured spending about 8% of their expenditures on outpatient care, while insured households spent 5%.

#### Catastrophic Health Expenditure

Table 3 presents the estimates of the incidence and intensity of CHE. Insured households had lower incidence of CHE compared to uninsured households. Only 7% of insured households incurred catastrophic costs as a result of healthcare compared to 12.3% of uninsured households. In terms of intensity of CHE (CHE overshoot), household health expenditure exceeded the 40% threshold by an average of 17% for uninsured households and 11% for insured households. Overall, 11.7% (203 households) of all (insured and uninsured) sampled households experienced catastrophic expenditure.

**Table 3:** Incidence and intensity of catastrophic health expenditure and household coping mechanisms

	**Uninsured** **%(n)**	**Insured** **%(n)**	**Total** **% (n)**
CHE Head Count	12.3(189)	7.0(14)	11.7(203)
CHE overshoot	17%	11%	16%

#### Proportion of households falling below the poverty-line before and after making OOP

Table 4 shows poverty rates before and after calculating OOP payments. The results show that 61.8% of individuals already lived below the national poverty line before paying for healthcare. After OOP payments, the poverty rate increased by 2.03 percentage points. The average deficit (that is, the poverty gap) to reach the poverty line of the population was Ksh 17,462(US$145) before taking OOP payments into account. After considering OOP payments, the average deficit increased to Ksh 18,378 (US$153).

**Table 4:** Poverty headcount before and after making OOP

	**Individuals falling below the poverty line before OOP**	**Individuals falling below the poverty line after OOP**
Uninsured Poverty headcount (%, n)	64.0(1003)	65.9 (1034)
Poverty Gap (KES, range)	17,744 (17,174–18,315)	18,682(18,054–19,310
Normalized poverty gap (%, range)	45.5% (44.0–46.9)	47.9% (46.3–49.5)
Insured Poverty headcount (%, n)	44.9(92)	47.3(97)
Poverty Gap (KES, range)	14,390 (12,440–16,340)	15,144 (12,951–17,338)
Normalized poverty gap (%, range)	36.9% (31.9–41.9)	38.8% (33.2–44.4)
Total Poverty headcount (%, n)	61.8(1095)	63.8(1,131)
Total Poverty Gap (KES, range)	17,462.52 (16,913.23–18,011.81)	18,378.36 (17,772.35–18,984.37)
Normalized poverty gap (%, range)	44.7% (43.5–46.2)	47.09% (45.54–48.64)

#### Factors associated with the incidence of Catastrophic Health Expenditure

Table 5 presents the results of logistic regression of the relationship between household characteristics and CHE incidence﻿. In adjusted analyses, poorer households, those that reported an outpatient visit in the last four weeks or were admitted to a health facility in the last 12 months had increased odds of CHE. Households with insurance and wealthier households were less likely to incur CHE (with health insurance-OR = 0.41, AOR = 0.40 and households in Q5 OR = 0.25, AOR = 0.42).

**Table 5:** Logistic Model estimating likelihood of incurring catastrophic health expenditure (*n* = 1773)

Characteristic	Unadjusted Estimates			Adjusted Estimates		
	**OR**^**a**^	**95% CI**^**a**^	***p*-value**	**OR**^**a**^	**95% CI**^**a**^	***p*-value**
**Age**						
18–24	0.33	(0.11, 0.79)	**0.024**	0.51	(0.15, 1.47)	0.2
25–44	0.29	(0.19, 0.45)	** < 0.001**	0.53	(0.28, 1.01)	0.052
45–64	0.53	(0.35, 0.80)	**0.003**	0.90	(0.53, 1.54)	0.7
˃64	—			—		
**Sex**						
Female	—			—		
Male	1.03	(0.72, 1.44)	0.9	1.54	(0.99, 2.38)	0.053
**Marital status**						
Divorced/separated	—			—		
Married/ living together	0.89	(0.49, 1.75)	0.7	1.06	(0.53, 2.30)	0.9
Never married/never lived together	0.82	(0.31, 2.04)	0.7	0.95	(0.33, 2.67)	> 0.9
Widowed	1.55	(0.82, 3.13)	0.2	0.88	(0.40, 2.04)	0.8
**Ever attended school**						
Yes	0.56	(0.39, 0.81)	**0.002**	0.90	(0.54, 1.52)	0.7
No	—			—		
**Have health insurance**						
Yes	0.41	(0.18, 0.80)	**0.017**	0.40	(0.17, 0.85)	**0.027**
No	—			—		
**Wealth quintile**						
Q1	—			—		
Q2	0.78	(0.50, 1.20)	0.3	0.90	(0.54, 1.48)	0.7
Q3	0.49	(0.29, 0.79)	**0.004**	0.55	(0.30, 0.98)	**0.044**
Q4	0.24	(0.12, 0.43)	** < 0.001**	0.34	(0.16, 0.70)	**0.004**
Q5	0.25	(0.13, 0.44)	** < 0.001**	0.42	(0.18, 0.96)	**0.044**
**Below poverty line**						
Yes	3.73	(2.56, 5.56)	** < 0.001**	3.14	(1.84, 5.51)	** < 0.001**
No	—			—		
**Member of financial group chama**						
Yes	1.03	(0.73, 1.44)	0.9	1.47	(0.96,2.22)	0.71
No	—			—		
**Admitted last 12 m**						
Yes	3.75	(2.31, 5.91)	** < 0.001**	3.18	(1.78, 5.55)	** < 0.001**
No	—			—		
**Outpatient visits last 4w**						
Yes	3.72	(2.94, 4.75)	** < 0.001**	4.09	(3.13, 5.39)	** < 0.001**
No	—					
**Presence of chronic ailment**						
Yes	1.98	(1.41, 2.81)	** < 0.001**	1.43	(0.94, 2.17)	0.10
No	—			—		

^a^*OR* Odds Ratio(natural odds), *CI* Confidence Interval

#### Financial burden of seeking healthcare

Most of the households described their health costs as high causing substantial burden on the household and resulting in major financial setbacks on the households. Cost of transport to health facilities contributed to the high cost of seeking care because patients often needed to be accompanied to health facilities, thereby doubling the cost of transport. One household respondent acknowledged the lurking financial risks in seeking healthcare even if one was not currently ill:

“*If you haven't gotten sick, it’s easy to say healthcare costs are low, but if you have ever been very sick the way I had started going to Eldoret [location of a national referral hospital] or if you get a chronic illness, it costs you a lot of money. Serious sickness –and I am not talking about simple diseases like malaria- requires lots of money*” IDI_C_Not_Active_02.

Respondents repeatedly expressed feelings of anxiety and despair in their ability to meet high medical costs. Those without health insurance reported feelings of being worried about falling sick and feeling financially strained because of healthcare costs.

“*My son is sickly and a month would not pass without us taking him back to the hospital. I have not started the treatment of my wife’s illness which I am sure will pull me down even more especially mentally. I keep wondering when will be able to take her for medical attention. I don’t know where I will start if we are referred to a bigger hospital.* IDI_B_Never_02.

Many of the rural households included extended family members and therefore had increase healthcare needs. Case study 1 further illustrates the burden of seeking healthcare for a large rural household.

**Table:** 

**Case study 1: Burden of seeking healthcare**
Evaline lives with her husband and 10 other family members including children, grandchildren and nieces. She and her husband suffer from chronic conditions. She has hypertension and painful arthritis on her knees. Her husband suffers from diabetes and has a chronic kidney condition. He has a wound on his leg that is taking long to heal because of his diabetes. Other health condition in the household include asthma, malaria, eye problems, pneumonia, and suspected mental illness. Evaline admits that she has used traditional medicine for her hypertension but also continues with her medicine. Despite having health insurance, the household spends a lot of money on seeking healthcare especially for her husband who depends on drugs for the kidney condition *“We use a lot of money and that’s why we are just here at home now, because we don’t have the money to go to hospital. When you go to hospital they want a lot of money. For example, if you say that you will go to [private health facility X] they want almost 4000 shillings, so when we have to, we just go to [public facility Y] even if you don’t get well, God will help you* Her firewood selling business doesn’t give them enough income and her husband cannot work because of his condition. On average, her hypertension medication costs Ksh. 600 monthly while her husband’s drugs cost Ksh. 3,000 monthly. The last time he was admitted, their son paid the bill of approximately ksh. 20,000 through fundraising *“You see when we are seeking treatment we need to give out money and that’s where most of the money goes. Sometimes you have nothing, you are just there. Will you tell your child to take you to hospital? Sometimes even your child has no money. The child might delay in taking you to hospital until the illness goes away on its own… If you are unable to go to hospital, what will you do? It will just go away on its own.”* (IDI_B_Active_01)

Households reported multiple financial strategies to cope with the costs of healthcare, including selling household assets and livestock. Some participants also reported closing their businesses and delaying other expenditures like improving their housing. For instance, one respondent had to sell iron sheets he had bought to complete his new brick house to pay for a medical bill. These set the families back financially. “*If I had taken that 47,000($390) shillings and invested it in a business, it would have helped me in some way. But now, it didn’t help me. Of course, it helped me because I was able to discharge my child. But it didn’t really help me develop.”* IDI_A_Not_Active_01. The other common means of meeting healthcare costs was through borrowing from friends and family, fundraising and taking loans. Elderly respondents reported relying on their children to pay their medical costs and health insurance premiums. One respondent however expressed the unreliability of raising funds through friends and relatives because they were also likely to be financial insecure.

#### Benefits of insurance

Some respondents with active NHIF membership shared positive experiences of having NHIF including feeling confident in their health insurance covering their healthcare costs:

“*What motivated me to go for it [NHIF] was that I felt it would help instead of holding fundraising. When you have a sick patient in hospital who requires about 30,000 or 20,000 shillings for treatment, that card can help you cover the costs so I will figure out a way to pay for it*.” IDI_Active_B_02.

Households reported that NHIF had helped clear some high medical bills as highlighted in the case below.

**Table:** 

**Case study household 2: Benefits of health insurance**
Maria is a 76-year-old female, widowed, currently incapacitated and reliant on her daughter in law to provide care. Maria suffers from arthritis, hypertension and ulcers. She has NHIF but has to buy medication out-of- pocket because the [public?] facilities do not have medication. However, her most recent inpatient bill of KSh. 16,000(US$130) was fully paid for by NHIF. Before enrolling for NHIF, her family had to fundraise to cover the cost of her care, which created many family conflicts. With NHIF coverage, family conflict has reduced and Maria’s son believes that having the insurance has helped Maria feel more cared for and appreciate her family more. (IDI_F_Active_01)

## Discussion

We found that a vast majority of rural households did not have health insurance (88.4%) exposing them to financial risk while seeking healthcare. Health insurance was not affordable for majority of households—both insured (60%) and uninsured (80%). Rural households spent an average of 12% of their household budget on OOP spending which was catastrophic to 11.7% of the households. While uninsured households experienced higher and more intense levels of CHE compared to the insured households, both insured and uninsured households reported high OOP spending and similar levels of impoverishments by OOP. Participants expressed concerns about value of health insurance coverage given its cost, services, and financial protection relative to other social and economic needs that they face on daily basis. Households resulted to borrowing, fundraising, taking short term loans from family and friends and sale of family assets in order to meet healthcare costs.

According to the Kenya Household health expenditure and utilization survey (KHHEUS) of 2018, 7.1% of the Kenyans incurred catastrophic healthcare payments while 7.9% of the rural population incurred CHE. The KHHEUS reported 46.9% of the population living below the poverty line in rural areas before OOP costs and rose to 49.1% when considering OOP costs [25] while our study reported 61.8% of the rural population living below the poverty and rose to 63.8% after OOP costs. The difference between our finding and the national survey finding could be explained by the fact that KHHEUS is based on a national sample with both urban and rural population and was last conducted in 2018 while our study conducted in 2021 focused on a rural population in Busia County. It is plausible that our finding of 11.7% of households experiencing CHE is part of the upward trend in the incidence of CHE. The 2018 KHHEUS survey showed that more Kenyans (7.1%) [26] experienced CHE compared to the previous 2007 survey (4.6%) [27]. Previous studies have shown higher, more severe incidence of OOP and CHE among the rural population compared to urban areas [25, 28]. The vulnerability of rural populations is further compounded by high poverty rates, reliance on small scale farming, effects of ecological factors like flooding and the economic effects of the COVID-19 pandemic reported in study setting. In addition, Busia county has one of the highest poverty rates in the country (66%) contributing to the unaffordability of healthcare among residents of the county [29, 30].

Informal labour in Kenya is not homogenous and workers vary in their ability to pay for healthcare. According to NHIF’s definition, informal workers refer to individuals who are unemployed, involved in casual labour or running small scale businesses [12]. Attempts to mandate health insurance requires strategies to address 2 main segments of the informal worker population i.e. those who lack the ability to pay for health insurance and those who can afford health insurance but chose not to enrol. According to our findings, NHIF is unaffordable to 60% of the uninsured informal workers. Although slightly lower, our findings are consistent with previous studies that found NHIF unaffordable to 75% of the population and findings of other willingness to pay studies in Kenya revealed that households were only willing to pay 300 per month compared to the Ksh. 500 charged [11, 31]. Among the insured, we found that NHIF was unaffordable to over 80% indicating that other more compelling drivers to enrolment despite unaffordability of premiums. Indeed, the presence of a chronic illness requiring frequent services as well as anticipated costly medical procedures were found to be the main drivers of enrolment consistent with findings of other studies on voluntary health insurance enrolment [32, 33]. Such adverse selection of members where high risk individuals enrol for health insurance limits risk cross-subsidization and destabilizes risk pools [34, 35].

Countries that have achieved greater coverage using mandatory insurance have fully subsidized the membership for the poor [1, 36]. Many of the rural households participating in our study displayed high levels of vulnerability but were not beneficiaries of social protection programs. Studies in LMIC have found that a small proportion (30%) of the vulnerable groups are enrolled into social health insurance programs [37, 38]. In Kenya, significant gaps in the implementation of the health insurance subsidy program were reported indicating that 65% of the beneficiaries of the health insurance subsidy belonged to the richest quintiles [11, 39]. More recently, the national indigent program rolled out registration of indigent households targeting 1 million households. The households included were identified from a list of vulnerable households provided by ministry of Labour and social services. There have been concerns however about the hastiness, inclusion process and the unclear strategies used to identify households to benefit from the national indigent programs. For health insurance subsidies to have the desired effect of extending coverage to the extremely poor, there is need to invest in robust national targeting mechanisms and framework of identification and verification of poor households [40].

Our study found that NHIF was unaffordable to 60% of the uninsured households, implying that 40% of the uninsured in Kenya can afford health insurance but only 11% of the population had enrolled. One of the reasons for low enrolment among those with the ability to pay, is the dissatisfaction with the NHIF cover. Respondents did not see the value of having NHIF because of unavailability of services in public facilities. Even though NHIF covers services in accredited public and private facilities, most rural communities have a limited network of private facilities [12]. Indeed, our findings reveal that inadequate coverage of health services in the NHIF benefit package and unavailability of services in public facilities results in high OOP among the insured as has been found in other studies [11, 41]. These inadequacies forces households to incur OOP by purchasing drugs and services in the private market. Forcing households who can afford to enrol in NHIF with no clear strategies for addressing health system weaknesses and increasing awareness on the NHIF benefit package would be unethical. Efforts to increase coverage and financial protection among this population will need to be accompanied by strategies to address weaknesses in the benefit package and delivery of quality health services at the facilities.

### Policy implications and recommendations

The need for financial risk protection among the vulnerable rural population is an important and urgent priority. Although mandating NHIF enrolment in Kenya in line with global debates advocating for compulsory contribution, it is challenging to enforce mandatory payments among the informal sector [3]. Mandatory insurance programs can only be enforced through payroll deduction for the employed or by imposing penalties for those who fail to comply [5]. Adequate caution needs to be taken in the enforcement of mandatory insurance to ensure that vulnerable households are not further penalised through such sanctions due to their inability to comply with compulsory enrolment into NHIF. The government should employ mechanisms to segment the informal worker population to differentiate those with ability to pay and those who cannot afford NHIF. Penalties should only be enforced among those with the ability to pay and are not enrolled in NHIF.

The government should prioritize rapidly rolling out the national subsidy program to cover poor households lacking the ability to pay for NHIF. The targeting of poor households should be strengthened to ensure the poor benefit from the programs. It is also important that those mandated to pay, are guaranteed access quality health services. This can be done by addressing the service delivery and supply chain issues that continue to drive OOP spending among the insured. Evidently, enforcing mandatory insurance for increased protection against OOP will not have desired benefits if the weaknesses in the benefit package and health service delivery are not addressed. The government therefore needs to prioritize broader health system reforms to improve health service delivery in public facilities. NHIF also needs to create adequate awareness on the services covered to ensure members are not charged for services that are covered by the fund. From our findings, adverse selection remains a threat to the NHIF scheme given the enrolment through health facilities. Because social health insurance cannot lock out members based on pre-existing conditions, enrolment NHIF needs to conduct extensive enrolment drives among the general population so as to include healthy members too.

### Strengths and limitations

Although many studies in Kenya have assessed the impoverishing effects of catastrophic healthcare payments, few have focused on the informal workers in rural areas. This is important given ongoing policy debates to increase coverage among informal worker households. Previous studies have also been limited methodologically as most approached the gap from a quantitative approach. Our study employed a mixed method approach that provides a more nuanced understanding of the challenges faced by rural households. However, a key limitation in our study is the low health insurance enrolment rates among the rural population which limited our ability to infer the level of protection NHIF offers among the insured because a small proportion of the population was covered by health insurance. In addition, our assessment of catastrophic payments and impoverishments only captures the negative effects on households that sought healthcare at the health facility. Although we appreciate the increased vulnerability of households not seeking care, our methodology did not estimate the effects on households that did not seek conventional care and is an area for future research. Lastly, household expenditure was obtained by asking the respondents to recall expenditure for specific periods. This may be prone to recall bias but effort was made to minimize bias by using different recall periods for different items depending on the frequency of consumption. For instance, the recall period for items bought frequently such as food items is limited to the last 7 days and one-year recall period for items bought less frequently like land and education.

## Conclusion

Rural populations have increased vulnerability to financial risk related to healthcare costs and lack adequate financial risk protection. While national reforms on NHIF illustrate commitment by the Kenyan government to the UHC agenda through mandatory NHIF membership, our findings showed that this commitment might be better informed by considering the affordability and extent of protection offered by NHIF. Not only is the NHIF premium unaffordable to majority of these households but the financial protection that it provides is inadequate to shield households against catastrophic healthcare payments because of inadequate benefit package and the unavailability of health services in public facilities. As such, it will be necessary to develop suitable strategies to ensure the inclusion of rural uninsured households who have limited ability to pay for healthcare and health insurance. Expansion of coverage will need to be accompanied by efforts to improve healthcare provision at public healthcare facilities. Therefore, to increase coverage among rural informal workers through mandatory enrolment and ensure that the vulnerable in Kenya and other LMICs settings are covered against catastrophic healthcare spending, there is need to simultaneously address affordability barriers to healthcare and health insurance enrolment and improve service coverage.

## Data Availability

Data will be made available upon request.
